# Comparison of the modified low-dose cytarabine and etoposide with decitabine therapy for elderly acute myeloid leukemia patients unfit for intensive chemotherapy

**DOI:** 10.18632/oncotarget.23629

**Published:** 2017-12-23

**Authors:** Seung-Hwan Shin, Byung-Sik Cho, Sung-Soo Park, Sung-Yeon Cho, Young-Woo Jeon, Jae-Ho Yoon, Seung-Ah Yahng, Sung-Eun Lee, Dong-Gun Lee, Ki-Seong Eom, Yoo-Jin Kim, Seok Lee, Chang-Ki Min, Seok-Goo Cho, Dong-Wook Kim, Jong-Wook Lee, Woo-Sung Min, Hee-Je Kim

**Affiliations:** ^1^ Department of Hematology, Yeouido St. Mary’s Hospital, College of Medicine, The Catholic University of Korea, Seoul, Republic of Korea; ^2^ Department of Hematology, Catholic Blood and Marrow Transplantation Center, Seoul St. Mary’s Hospital, Leukemia Research Institute, College of Medicine, The Catholic University of Korea, Seoul, Republic of Korea; ^3^ Department of Infectious Disease, Seoul St. Mary’s Hospital, The Catholic University of Korea, Seoul, Republic of Korea; ^4^ Department of Hematology, Incheon St. Mary’s Hospital, College of Medicine, The Catholic University of Korea, Seoul, Republic of Korea

**Keywords:** elderly acute myeloid leukemia, modified low-dose cytarabine, etoposide, decitabine, low-intensity chemotherapy

## Abstract

To overcome unsatisfactory results of classical low-dose cytarabine (LDAC) of cytarabine ≤20 mg twice daily (BID) subcutaneously for 10 days for patients with elderly acute myeloid leukemia (eAML), we evaluated a modified LDAC (mLDAC) of cytarabine 20 mg/m^2^ BID subcutaneously plus etoposide 50 mg BID orally for 14 days. To determine its feasibility, we compared outcomes of 77 and 42 eAML patients who received, respectively, mLDAC and decitabine (DAC; 20 mg/m^2^ intravenously daily for 5 days), which has shown better outcomes compared to those of classical LDAC. Most of baseline characteristics of two groups were well balanced. The mLDAC group had a higher complete response (CR) rate compared to the DAC group (46.8% vs. 19.0%, *P* < 0.01). Unlike the classical LDAC, mLDAC induced CR in patients with adverse cytogenetics, with its rate similar to that of the DAC group (33.3% vs. 20.0%; *P* = 0.58). Meanwhile, mucositis, neutropenic fever and invasive aspergillosis were more frequently observed in the mLDAC group, with no difference in early mortality between two groups (*P* > 0.05). The median overall survival rates of the mLDAC and DAC groups were comparable (8.7 vs 8.3 months, respectively, *P* = 0.35), presumably because the advantage of higher CR rate in the mLDAC group was offset by beneficial effects of marrow response, which is observed dominantly in the DAC group. Our results suggested that the outcomes of classical LDAC could be improved by modest modifications, to be comparable to those of DAC.

## INTRODUCTION

Acute myeloid leukemia (AML) is a hematologic malignancy most frequently affecting the elderly. As reported by the Surveillance, Epidemiology, and End Results (SEER) database, half of newly diagnosed patients are ≥65 years of age [1]. Survival rate of patients tends to be worse disproportionately to age at diagnosis [2], seemingly due to several patient-related factors, including poor performance status (PS) and comorbidities, or disease-related factors, including a high frequency of adverse cytogenetic risk, unfavorable molecular alterations, and multidrug resistance of the leukemia cells, features of elderly AML (eAML; commonly defined as AML in patients aged ≥60 years) [3]. Intensive chemotherapy (ICTx) should be performed preferentially for eAML patients, if they are expected to be tolerate it, considering the better outcomes achieved with ICTx compared with low-intensity treatments or best supportive care (BSC) in previous studies [4, 5]. Nevertheless, ICTx is contraindicated for a substantial proportion of patients because of a high probability of early mortality associated with poor PS or comorbidities.

A classical low-dose cytarabine regimen (cLDAC), consisting of cytarabine ≤20 mg subcutaneously (SC) twice daily (BID) for 10 days every 4–6 weeks, had been considered as an available therapeutic option for eAML patients unfit for ICTx [6–9]. Although a randomized trial showed better results compared with those of cytoreductive therapy using hydroxyurea, complete remission (CR) and overall survival (OS) rates were still unsatisfactory [6]. Among several efforts to develop novel drugs for these patients, hypomethylating agents (HMAs) have been receiving a lot of attention owing to its favorable toxicity profile [7–13]. Although randomized trials showed some benefits of decitabine (DAC) and azacytidine (AZA), including more potent anti-leukemic activity compared with treatments choice (cLDAC or BSC) or conventional care regimens (ICTx, cLDAC or BSC) [12, 13], the U.S. Food and Drug Administration did not approve either HMA for patients with eAML, except if they have oligoblastic (<30% of blasts) disease, because of a lack of definite survival advantages. Nevertheless, HMAs are preferred for this group of patients, owing to the better response and longer trend of survival compared with cLDAC.

As an alternative method to improve outcomes of eAML patients unfit for ICTx, we used a modification of cLDAC (mLDAC) involving extended administration of a relatively increased-dose of cytarabine plus etoposide, based on a number of previous observations, including the achievement of a relatively high CR rate by 20 mg/m^2^ BID cytarabine regimen, the benefit of a prolonged etoposide schedule, and their syngeneic anti-leukemic activity [14–18]. Our recent study showing an improved response and prolonged survival compared with those of previous reports of cLDAC suggested that this approach might be a feasible option [19]. In the current study, including eAML patients (age of ≥65 years) unfit for ICTx, we compared outcomes of consecutive patients who received mLDAC and DAC to investigate the degree of improvement in outcomes of cLDAC by this modest modification.

## RESULTS

### Patient demographics and baseline clinical characteristics

Of 119 eAML patients unfit for ICTx at our institution between October 2002 and December 2015, 77 (64.7%) received mLDAC and 42 (35.3%) received DAC as a first-line treatment. In the entire cohort, the median age of patients at diagnosis was 71 (range, 65–83) years, with 55 (46.2%) being ≤70 years. A high Eastern Cooperative Oncology Group (ECOG) PS score (defined as ≥2) and hematopoietic cell transplant co-morbidity index (HCT–CI; defined as ≥3) were observed in 59 (49.6%) and 42 (35.3%) patients, respectively. The median peripheral blood (PB) white blood cell count was 8.9 × 10^9^/l (range, 0.7–449.0 × 10^9^/l), with a median 28.0% (range, 0–97%) blast count. An initial median bone marrow (BM) blast count was 81.0% (range, 7.0–99.0%), with 105 patients (88.2%) having ≥30%. A myelodysplasia-related change or preceding hematologic diseases was observed in 23 patients (19.3%). In terms of cytogenetics risk, 112 (85.7%) and 17 (14.3%) patients had a favorable / intermediate, and adverse risk, respectively. The characteristics of patients, including the proportion of patients >70 years of age (51.9% vs. 51.7%, *P* = 0.73), or having a high HCT-CI (≥3; 33.8% vs. 38.1%, *P* = 0.64), and adverse cytogenetic risk (15.6% vs. 11.9%, *P* = 0.78), were comparable between the mLDAC and DAC group (*P* > 0.05), except significantly different proportions of patients with a low platelet count (<50 × 10^9^/l; 59.7% vs. 38.1%, *P* < 0.04), a high lactate dehydrogenase (above upper normal limit; 85.7% vs. 66.7%, *P* = 0.03), and treatment before September 2013 (93.5% vs. 4.8%, *P* < 0.01). In the propensity-score matching cohort, the characteristics of all 84 patients were not significantly different between the two groups (*P* > 0.10), apart from the proportion of patients who received treatment before September 2013 (92.9% vs 4.8%; *P* < 0.01). All baseline demographic and clinical characteristics of the entire cohort and propensity-score matching cohort were described in Table 1.

**Table 1:** Comparison of patients’ demographics and baseline clinical characteristics between the mLDAC and DAC groups

Characteristics	Entire cohort			Propensity score–matching cohort		
	mLDAC group	DAC group	*P*	mLDAC group	DAC group	*P*
Number of patients	77	42		42	42	
Age at diagnosis, median (range)	71 (65–83) yrs	71 (65–83) yrs	0.43	71 (65–83)	71 (65–83)	0.78
≤70 yrs/>70 yrs	37 (48.1%)/40 (51.9%)	18 (42.9%)/24 (57.1%)	0.73	20 (47.6%)/22 (52.4%)	18 (42.9%)/24 (57.1%)	0.83
Sex						
Male/Female	41 (53.2%)/36 (46.8%)	21 (50.0%)/21 (50.0%)	0.88	23 (54.8%)/19 (45.2%)	21 (50.0%)/21 (50.0%)	0.83
ECOG PS score						
<2/≥2	41 (53.2%)/36 (46.8%)	19 (45.2%)/23 (54.8%)	0.40	21 (50.0%)/21 (50.0%)	19 (45.2%)/23 (54.8%)	0.83
HCT-CI						
<3/≥3	51 (66.2%)/26 (33.8%)	26 (61.9%)/16 (38.1%)	0.64	27 (64.3%)/15 (35.7%)	26 (61.9%)/16 (38.1%)	1.00
Disease etiology						
*De novo*/MRC or secondary	65 (84.4%)/12 (15.6%)	31 (73.8%)/11 (26.2%)	0.25	33 (78.6%)/9 (21.4%)	31 (73.8%)/11 (26.2%)	0.80
WBC count, median (range)	8.9 (0.7–449.0) × 10^9^/l	8.8 (1.0–272.0) × 10^9^/l	0.70	6.0 (0.8–325.6) × 10^9^/l	8.8 (1.0–272.0) × 10^9^/l	0.54
<10.0 × 10^9^/l/≥10.0 × 10^9^/l	40 (51.9%)/37 (48.1%)	23 (54.8%)/19 (45.2%)	0.92	24 (57.1%)/18 (42.9%)	23 (54.8%)/19 (45.2%)	1.00
Hemoglobin, median (range)	8.0 (3.2–14.3) g/dl	8.3 (4.2–11.2) g/dl	0.91	8.3 (4.2–11.2) g/dl	8.3 (4.2–11.2) g/dl	0.65
≤8.0 g/dL/>8.0 g/dL	39 (50.6%)/38 (49.4%)	18 (42.9%)/24 (57.1%)	0.53	19 (45.2%)/23 (54.8%)	18 (42.9%)/24 (57.1%)	1.00
Platelet count, median (range)	41.0 (7.0–399.0) × 10^9^/l	65.5 (5.0–403.0) × 10^9^/l	0.01	46.0 (7.0–360.0) × 10^9^/l	65.5 (5.0–403.0) × 10^9^/l	0.20
<50 × 10^9^/l/≥50 × 10^9^/l	46 (59.7%)/31 (40.3%)	16 (38.1%)/26 (61.9%)	0.04	23 (54.8%)/19 (45.2%)	16 (38.1%)/26 (61.9%)	0.19
PB blast, median (range)	31.0 (0–97.0) %	16.5 (0–94.0) %	0.36	29.5 (0–97.0%)	16.5 (0–94.0) %	0.70
<30 %/≥30%	37 (48.1%)/40 (51.9%)	25 (59.5%)/17 (40.5%)	0.31	21 (50.0%)/21 (50.0%)	25 (59.5%)/17 (40.5%)	0.51
BM blast, median (range)	82.0 (7.0–99.0) %	73.5 (20.0–99.0) %	0.25	80.0% (20.0–99.0%)	73.5 (20.0–99.0) %	0.81
<30 %/≥30%	10 (13.0%)/67 (87.0%)	4 (9.5%)/38 (90.5%)	0.79	7 (16.7%)/35 (83.3%)	4 (9.5%)/38 (90.5%)	0.52
LDH						
≤UNL/>UNL	11 (14.3%)/66 (85.7%)	14 (33.3%)/28 (66.7%)	0.03	8 (19.0%)/34 (81.0%)	14 (33.3%)/28 (66.7%)	0.22
Cytogenetic risk						
Favorable or Intermediate/Adverse	65 (84.4%)/12 (15.6%)	37 (88.1%)/5 (11.9%)	0.79	38 (90.5%)/4 (9.5%)	37 (88.1%)/4 (11.9%)	1.00
Years at mLDAC or DAC^*^						
Before Oct 2013/After Oct 2013	72 (93.5%)/5 (6.5%)	2 (4.8%)/40 (95.2%)	<0.01	39 (92.9%)/3 (7.1%)	2 (4.8%)/40 (95.2%)	<0.01

mLDAC = modified low-dose cytarabine; DAC = decitabine; ECOG PS = the Eastern Cooperative Oncology Group performance status; HCT-CI = the hematopoietic cell transplant co-morbidity index; MRC = myelodysplasia-related change; PB = peripheral blood; BM = bone marrow; LDH = lactate dehydrogenase; UNL = upper normal limit.

^*^Since Oct 2013, DAC was added as an available option for elderly acute myeloid leukemia patients unfit for intensive chemotherapy at out institution.

### Response rates

Patients received a median of 2 (range, 1–8) cycles of mLDAC or 4.5 (range, 1–14) cycles of DAC. After a median 1 (range, 1–2) and 4 (range, 1–7) cycles, respectively, 39 patients, including 3 incomplete CR (CRi) and 1 CR without platelet recovery (CRp) in the mLDAC group and 10 patients, including 2 CRp, in the DAC group achieved composite CR (CRc). The CR and CRc rates of the mLDAC group were significantly higher than those of the DAC group (46.8% vs. 19.0%, *P* < 0.01; and 50.6% vs. 23.8%, *P* < 0.01, respectively). In univariate analysis, age (≤70 yrs vs. >70 yrs) and HCT–CI (<3 vs. ≥3) were the only additional potential predictors affecting CR and CRc rates (45.5% vs. 29.7%, *P* = 0.08; and 46.8% vs. 31.0%, *P* = 0.09), respectively (Supplementary Table 1). Multivariate analysis showed that therapeutic regimen (mLDAC vs. DAC) was the only significant factor affecting the CR and CRc rate (odds ratio [OR] 3.72, 95% confidence interval [CI] 1.51–9.15, *P* < 0.01; and OR 3.28, 95% CI 1.40–7.65, *P* = 0.01), with an only trend of age (≤70 vs. >70 years) affecting the CR rate (OR 1.96, 95% CI 0.90–4.30, *P* = 0.09) (Table 2). In 17 patients with adverse cytogenetic risk, 3 in the mLDAC group and 1 in the DAC group achieved CR (33.3% vs. 20.0%, *P* = 0.58). When we analyzed patients in the propensity-score matching cohort, the CR and CRc rates of the mLDAC group also were significantly higher than those of the DAC group (50.0% vs. 19.0%; *P* = 0.01 and 52.4% vs. 23.8%; *P* = 0.01, respectively).

**Table 2:** Univariate and multivariate analyses of factors affecting CR, CRc, OR and OS rates

Factors	Univariate analysis	*P*	Multivariate analysis	*P*
	Rate		Odds or hazard ratio (95% CI)	
***CR rate***				
Therapeutic regimen				
mLDAC vs. DAC	46.8% vs. 19.0%	<0.01	3.72 (1.51–9.15)	<0.01
Age at diagnosis				
≤70 yrs vs. >70 yrs	45.5% vs. 29.7%	0.08	1.96 (0.90–4.30)	0.09
***CRc rate***				
Therapeutic regimen				
mLDAC vs. DAC	50.6% vs. 23.8%	<0.01	3.28 (1.40–7.65)	0.01
HCT-CI				
<3 vs. ≥3	46.8% vs. 31.0%	0.09	1.95 (0.86–4.43)	0.11
***OR rate***				
Therapeutic regimen				
mLDAC vs. DAC	55.8% vs. 40.5%	0.11	1.83 (0.84–3.99)	0.13
Age at diagnosis				
≤70 yrs vs. >70 yrs	61.8% vs. 40.6%	0.02	2.34 (1.11–4.93)	0.03
***OS rate (at 1 yr)***				
Therapeutic regimen				
mLDAC vs. DAC	44.2% vs. 40.7%	0.35	0.79 (0.50–1.25)	0.31
Age at diagnosis				
≤70 yrs vs. >70 yrs	55.5% vs. 32.5%	<0.01	0.49 (0.32–0.74)	<0.01
HCT-CI				
<3 vs. ≥3	51.3% vs. 41.3%	0.05	0.76 (0.42–1.29)	0.06
Cytogenetic risk				
Non-adverse vs. adverse	46.4% vs. 25.5%	0.10	0.65 (0.42–1.02)	0.29

CI = confidence interval; CR = complete remission; mLDAC = modified low-dose cytarabine; DAC = decitabine; HCT-CI = the hematopoietic cell transplant co-morbidity index; CRc = composite complete remission; OR = overall response; OS = overall survival.

^*^In this table, only factors with *P* < 0.10 in univariate analysis are shown, with the exception of the therapeutic regimen (mLDAC vs. DAC) which is the main area of interest in this study.

**Figure 1:**
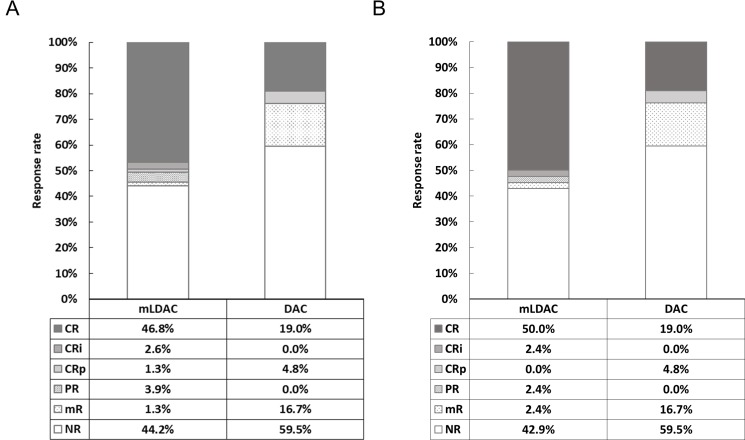
Detailed response rates of the mLDAC and DAC groups, in (**A**) the entire cohort and (**B**) propensity-score matching cohort

In terms of the overall response (ORR) rate, no significant difference between the mLDAC and the DAC groups was observed (55.8% vs. 40.5%, *P* = 0.11). Age (≤70 vs. >70 years) was the only potential factor affecting the ORR rate (52.7% vs. 35.9%, *P* = 0.07) in univariate analysis (Supplementary Table 1). Multivariate analysis also showed that age was the only significant factor affecting the ORR (OR 2.34, 95% CI 1.11–4.93; *P* = 0.03) (Table 2). When we analyzed patients in the propensity-score matching cohort, the ORR rate also was not significantly different between the mLDAC and DAC groups (55.8% vs. 40.5%, *P* = 0.11). The detailed response rates of two groups of the entire cohort and propensity-matching cohort are described in Figure 1.

### Toxicities and early mortality

Although the incidences of grade 3–4 oral mucositis (44.2% vs. 9.5%, *P* < 0.01), neutropenic fever (74.0% vs. 38.1%, *P* < 0.01), and invasive aspergillosis (16.9% vs. 2.4%, *P* = 0.02) were significantly higher in the mLDAC group, early mortality rates on day 30 of the two groups were comparable (13.0% vs. 9.5%, *P* = 0.77). When we analyzed patients in the propensity-score matching cohort, the incidences of grade 3–4 toxicities, including mucositis (45.2% vs. 11.9%; *P* < 0.01) and neutropenic fever (73.8% vs. 38.1%; *P* < 0.01) were significantly higher in the mLDAC group. However, the incidence of grade 3–4 invasive aspergillosis of the mLDAC group showed only a high trend compared to that of the DAC group (16.7% vs. 2.4%; *P* = 0.06). Early mortality rates on day 30 in the two groups were also not significantly different (11.9% vs. 9.5%; *P* = 1.00). The incidences of other relevant grade 3–4 relevant toxicities, including nausea/vomiting, diarrhea, hepatotoxicity, and renal toxicity, were not significantly different (*P* > 0.05) in both the entire and propensity-score matching cohorts, as shown in Table 3.

**Table 3:** Comparison of clinically relevant grade 3–4 toxicities between the mLDAC and DAC groups

Toxicities	Entire cohort			Propensity score-matching cohort		
	mLDAC group	DAC group	*P*	mLDAC group	DAC group	*P*
Oral mucositis	31.2%	9.5%	0.01	45.2%	9.5%	<0.01
Neutropenic fever	74.0%	38.1%	<0.01	73.8%	38.1%	<0.01
Invasive fungal infection	16.9%	2.4%	0.02	16.7%	2.4%	0.06
Nausea/vomiting	9.1%	4.9%	0.49	9.5%	4.9%	0.68
Diarrhea	5.2%	2.4%	0.66	7.1%	2.4%	0.62
Hepatotoxicity	2.6%	2.4%	0.99	2.4%	2.4%	1.00
Renal toxicity	5.2%	2.4%	0.66	4.8%	2.4%	1.00

mLDAC = modified low-dose cytarabine; DAC = decitabine.

### Relapse or disease progression

Of 60 patients (43 in the mLDAC group and 17 in the DAC group) who achieved ORR, 41 (29 in the mLDAC group and 12 in the DAC group) experienced relapse or disease progression. The median CR and CRc duration of the mLDAC group was relatively longer than that of the DAC group, although the difference was not significant (22.9 vs. 10.7 months, *P* = 0.23 and 24.3 vs. 10.0 months, *P* = 0.05). However, the median ORR duration of the mLDAC group was significantly longer than that of the DAC group (22.7 vs. 9.3 months; *P* = 0.04).

### Salvage treatments

After receiving mLDAC or DAC, 100 patients (63 in the mLDAC group and 37 in the DAC group) experienced refractoriness, relapse, or disease progression. Of these patients, 18 (28.6%) of the mLDAC group and 13 (35.1%) of the DAC group received salvage chemotherapies, including 18 (6 in the mLDAC group and 12 in the DAC group) who received mLDAC and 13 (12 in the mLDAC group and 1 in the DAC group), received abbreviated (reduced-dose and/or shortened-duration) ICTx. Of note, 7 patients (58.3%) of the DAC group, who received mLDAC as a salvage regimen, achieved subsequent ORR, including 5 CR, 1 CRi, and 1 marrow response (mR), whereas none of the mLDAC group, who received additional mLDAC as a salvage regimen, achieved significant responses. Of those who received abbreviated ICTx, 6 (50.0%) of the mLDAC group and 1 (100%) of the DAC group achieved subsequent CRc, including 1 CRi and 2 CRp (Supplementary Tables 2 and 3).

### Overall survival

**Figure 2:**
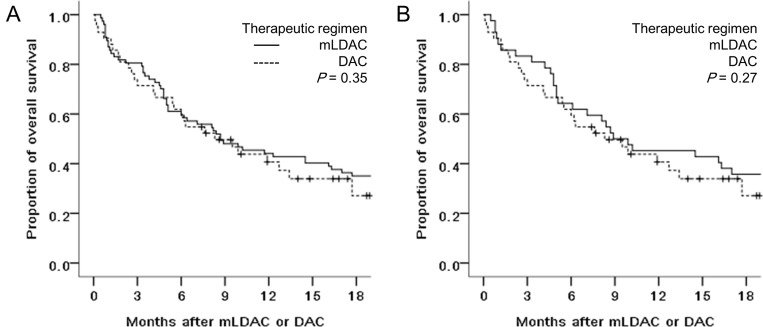
Comparison of OS rates between the mLDAC and the DAC groups, in (**A**) the entire cohort and (**B**) propensity-score matching cohort.

The OS rates of the mLDAC and DAC groups were not significantly different (44.2% vs. 40.7% at 1 year, *P* = 0.35), with a median OS of 8.7 and 8.3 months, respectively (Figure 2). In univariate analysis, age (<70 vs. ≥70 years), HCT-CI (<3 vs. ≥3), and cytogenetic risk (non-adverse vs. adverse) were potential predictors affecting the OS rate (55.5% vs. 32.5% at 1 year, *P* < 0.01, 51.3% vs. 41.3% at 1 year, *P* = 0.05, and 46.4% vs. 25.5% at 1 year, *P* = 0.10, respectively) (Supplementary Table 1). Multivariate analysis showed that age (<70 vs. ≥70 years) was the only significant factor affecting the OS rate (hazard ratio [HR] 0.49, 95% CI 0.32–0.74, *P* < 0.01), with HCT-CI (<3 vs. ≥3)showing only a trend of affecting the OS rate (HR 0.76, 95% CI 0.42–1.29; *P* = 0.06) (Table 2). When we analyzed patients in a propensity-score matching cohort, no significant difference of OS rates also was observed between the mLDAC and DAC groups (45.2% vs. 40.7% at 1 year; *P* = 0.27).

### Subgroup analyses

In the respective analyses for the mLDAC and the DAC group, age (≤70 vs. >70 years) was the only potential predictor affecting CR (33.3% vs. 8.3%, *P* = 0.06) and CRc (38.9% vs. 12.5%, *P* = 0.07) rates in the DAC group. Potential predictors of ORR rates were HCT-CI (<3 vs. ≥3) in the mLDAC group (62.7% vs. 42.3%, *P* = 0.09) and age (≤70 vs. >70 years) in the DAC group (51.6% vs. 9.1%, *P* = 0.07). Age (≤70 vs. >70 years) in both groups (51.4% vs. 37.5% at 1 year, *P* = 0.03; and 63.6% vs. 23.8% at 1 year; *P* = 0.01, respectively), and ECOG PS score (<2 vs. ≥2) and HCT-CI (<3 vs. ≥3) in the mLDAC group (48.8% vs. 38.9%, *P* = 0.03; and 51.0% vs. 30.8%, *P* = 0.07) were potential predictors affecting the OS rate (Supplementary Table 4). Multivariate analysis showed that age (≤70 vs. >70 years) was the only significant factor affecting the OS rate (HR 0.54, 95% CI 0.33–0.89; *P* = 0.02) in the mLDAC group.

**Figure 3:**
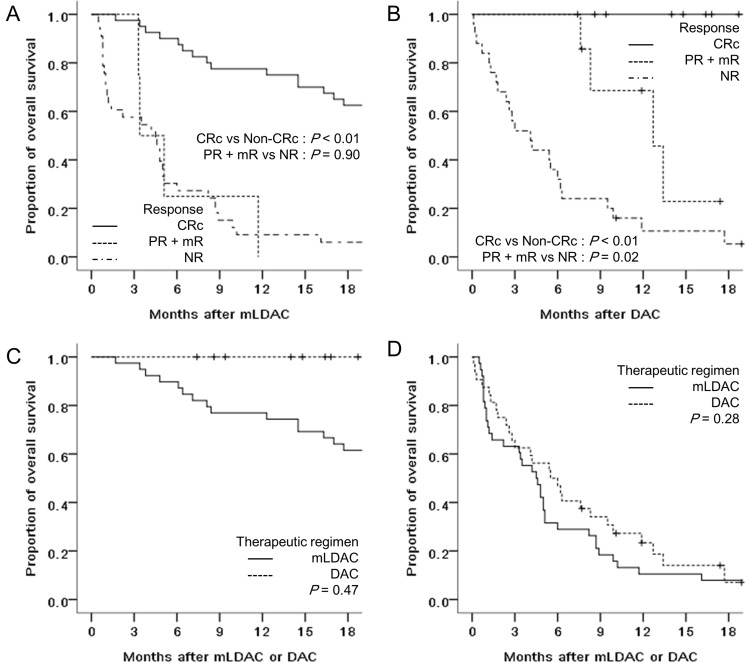
Comparison of OS rates between patients who achieved CRc and non-CRc of (**A**) the mLDAC, and (**B**) the DAC groups, and between the mLDAC and the DAC groups of the patients who achieved (**C**) CRc and (**D**) non-CRc.

Regarding the relationship between response and survival, the OS rates of patients who achieved CRc were significantly higher than the OS rates of those who did not in both the mLDAC group (76.9% vs. 10.5% at 1 year, *P* < 0.01) and the DAC group (100% vs. 18.7% at 1 year; *P* < 0.01) (Figure 3A and 3B). Meanwhile, there was no significant difference in OS rate between the patients who achieved CR and those who achieved CRc (79.4% vs. 75.0% at 1 year, *P* = 0.17). The OS rate of patients who achieved partial response (PR) or mR compared with that of those who had no response (NR) was significantly higher in the DAC group (68.6% vs. 10.7% at 1 year, *P* = 0.02), but not in the mLDAC group (0% vs. 11.8% at 1 year, *P* = 0.90) (Figure 3A and 3B). In addition, there was no significant difference in OS rates in patients achieving CRc (76.9% vs. 100% at 1 year, *P* = 0.47) and those not achieving CRc (10.5% vs. 23.4% at 1 year, *P =* 0.28) between the mLDAC and DAC group (Figure 3C and 3D). The OS rates of patients who relapsed or had disease progression were also not significantly different between the mLDAC and the DAC group (24.1% vs. 0% at 1 year, *P* = 0.61).

## DISCUSSION

The outcomes of eAML patients who received cLDAC in previous reports [6–8], were unsatisfactory, as indicated by a median OS of ≤6 month. To overcome these challenges, many novel agents have been investigated [7–12]. Since several pivotal studies, including the DACO-016 and AZA-AML-001 study, which showed improved outcomes of HMAs compared with conventional therapeutic modalities [12, 13], they have been preferred options in many countries [20]. Based on our previous report which suggested that mLDAC could be a feasible option [19], we expected that it also could be an alternative option for eAML patients unfit for ICTx, who are expected to have comparable outcomes with HMAs. In this study, we compared the outcomes of patients who were treated with mLDAC or DAC, which showed that modest modifications of cLDAC could not only enhance response rates, but also increase survival of the mLDAC group to be comparable to the DAC group.

Despite different CR rates of eAML patients who received cLDAC in several studies, most did not exceed 20% [6–8]. Meanwhile, the CR rate of patients in a study adopting 20 mg/m^2^ BID cytarabine for 10 days approached 30% [14], implying the possible benefits of an increased cytarabine dose. Regarding etoposide, a randomized trial by Zhang *et al.* which compared the outcomes of relapsed/refractory (RR) AML patients who received a low-dose CAG regimen (granulocyte colony-stimulating factor 20 μg/m^2^ for 14 days, aclarubicin 14 mg/m^2^ for 4 days, and cytarabine 10 mg/m^2^ BID for 14 days) with and without etoposide (14 mg/m^2^ for 14 days), showed that the etoposide group achieved a higher CR rate (71.1% vs. 50.9%; *P* < 0.01) [15]. Other studies suggested that a combination with cytarabine and etoposide potentiated anti-leukemic activity [16, 17] and a prolonged schedule for 14–21 days, which was adopted in this study, was more effective than the standard schedule for 3–5 days [18]. In line with these observations, the CR rate of the mLDAC group was relatively higher than that of patients receiving cLDAC or DAC in other studies [6–8, 12, 13]. Of note, in contrast to cLDAC of previous reports showing no efficacy in patients with adverse cytogenetic risk [6–8], mLDAC induced a significant response in this group of patients, which resulting in no significant difference in the CR rate between the mLDAC and the DAC groups. These results suggest that the anti-leukemic effect could be improved by modest modifications of cLDAC, even in patients with adverse disease-related features.

In this study, despite a higher CRc rates in the mLDAC group, there was no significant difference in OS rate between the mLDAC and the DAC groups. One reason explaining this is that a substantial proportion of patients in the DAC group, unlike the mLDAC group, achieved mR, which may contribute to their prolonged OS. In a subgroup analysis of the DAC group, patients who achieved mR had more survival benefits compared with those who had NR, which is consistent with a post-hoc analysis of a randomized phase III trial (AZA-001), showing that not only CR, but also PR and mR after HMA treatment as a best response, translated to survival advantages [21]. Although recent phase III trials of novel low-dose chemotherapy (LCTx) regimens (cLDAC vs cLDAC plus gemtuzumab ozogamicin and cLDAC vs. clofarabine) showed that a higher CRc rate in groups with novel agents did not result in improved OS rate, owing to the inferior post-relapse survival in the cLDAC groups [9, 22], post-relapse or disease progression survival were not different between two groups in our cohort, which implies the beneficial effect of mR for OS rate in the DAC group.

A representative merit of DAC is its favorable toxicity profile. The DACO-016 study showed that DAC was associated with a relatively low incidence of grade 3–4 toxicities, including neutropenic fever (34%), pneumonia (25%), and septic shock (6%), with a low rate of drug discontinuation owing to adverse events (6%) [13]. In another phase II study, that analyzed the efficacy and safety of DAC for eAML patients, the incidence of grade 3–4 neutropenia (9.5%), pneumonia (13.1%), sepsis (6.0%), and invasive aspergillosis (3.6%) was clinically acceptable [23]. The low incidence of grade 3–4 toxicities, including neutropenic fever and invasive aspergillosis, of the DAC group in this study also supports the good tolerability of DAC. In contrast, a higher proportion of patients in the mLDAC group experienced grade 3–4 neutropenic fever and invasive aspergillosis. Their incidences were significantly higher compared to not only patients of the DAC group, but also those who received cLDAC in previous reports [6–8]. However, the rates of early mortality were similar between the two groups, suggesting the feasibility of mLDAC. Nevertheless, strategies to reduce the frequency of infectious complications in patients who receive mLDAC are needed. A retrospective study of eAML patients who received LTCx, by Bainschab *et al.* [24], suggested that routine prophylactic antibiotic administration was associated with a low incidence of infectious complications. In a randomized phase III study, a prophylactic use of posaconazole, compared with intraconazole or fluconazole led to a significantly lower rate of mortality caused by fungal infections (2% vs. 5%, *P* < 0.01) and a lower incidence of invasive aspergillosis (1% vs. 7%, *P* < 0.01), in patients who experienced prolonged neutropenia after chemotherapy [25]. A high incidence of infection-associated toxicity in the mLDAC group may be overcome by using a more potent prophylaxis, which is being studied by our group.

Most patients with RR AML have an extremely poor prognosis with a long-term sustained CR rate of ≤5% resulting from chemotherapy only [26–29]. Several large retrospective studies showed that OS rates at 2 years did not exceed 15%, in those categorized as high-risk group owing to old age, short CR duration, and adverse cytogenetic risk [27, 28]. Considering the possibility of harboring these high-risk features, therapeutic strategies for most eAML patients who failed in their first-line treatment or relapsed have not been focused on cure following achievement of CR, but only on palliative control [26]. Of note, in this study, a substantial proportion of patients achieved additional CR after subsequent cycles of mLDAC after DAC failures. The epigenetic priming effect of DAC, which was observed in several previous studies [30, 31], may be an explanation. Considering the few reports of optimal strategies for RR eAML [26], a possible role of mLDAC as an attractive salvage regimen should be further investigated at least if patients received DAC as a first-line treatment.

In this study, the survival outcome of the mLDAC group was at least not inferior to that of the DAC group, with a higher CR rate, but more frequent infectious complications. It was also validated by the propensity-score matching cohort to alleviate any confounding effects from several unbalanced characteristics between the two groups. Improved outcomes of mLDAC compared with cLDAC in previous reports, coming close to outcomes achieved with DAC, a preferred option for eAML patients unfit for ICTx, suggest a modest modification of cLDAC could improve outcomes in this group of patients. The difference in the administration period between the two groups, arising from more recent usage of DAC, may complicate the interpretation of our results, because advances in supportive care over a decade might alter treatment outcomes. However, this does not change our overall conclusion, because the difference in their periods would favor outcomes in the DAC group. Considering its relatively low cost and easy accessibility, mLDAC may be an attractive therapeutic option for eAML patients unfit for ICTx. However, prospective comparative studies are needed to draw a definitive conclusion, considering the limitations of our study, including its retrospective nature, with no direct comparison between mLDAC and DAC. In addition, further efforts are also needed to determine the roles of more potent prophylaxis against infectious complications in mLDAC and mLDAC as a salvage regimen for patients experiencing DAC failure.

## MATERIALS AND METHODS

### Study population

At our institution, eAML received ICTx or LCTx according to a risk stratification strategy [19]. Since October 2002, patients determined to be candidates for LCTx by an ECOG PS score and/or HCT-CI of ≥2 have been treated with mLDAC [19]. In addition, DAC has been another option as LCTx for those ≥65 years of age, following the extended coverage of the National Health Insurance Service of Korea in October 2013. In this study, all consecutive patients ≥65 years of age who received LCTx, including mLDAC and DAC, were analyzed to determine whether modifications of cLDAC by extended administration of a relatively increased-dose of cytarabine plus etoposide could improve the outcomes of eAML patients unfit ICTx, compared with those of DAC. This retrospective study was approved by the Institutional Review Board of the Catholic University of Korea (KC17OESI0285).

### Low-intensity chemotherapy regimens

Patients of the mLDAC group received cytarabine (20 mg/m^2^ BID SC) plus etoposide (50 mg orally BID), every 6–8 weeks. If they achieved CRc after the first 1–2 cycles of mLDAC for 14 days, additional cycles of mLDAC for 10 days (maximum of 7 cycles) were administered if patients tolerated the treatment and relapse did not occur. Patients of the DAC group received DAC (20 mg/m^2^ intravenously daily) every 4 weeks until no response after the first 4–6 cycles, relapse, or disease progression, if they did not experience any unacceptable toxicity [13]. A BM aspiration and biopsy for assessment of response and disease progression was performed after every cycle of mLDAC before achieving CRc and after the second and fourth cycle of DAC and then every 3 cycles or as clinically indicated.

If patients failed to achieve a significant response or experienced relapse and disease progression, considering the individual clinical situation, they received a salvage chemotherapy consisting of additional mLDAC, abbreviated ICTx, or BSC.

### Definitions

Responses after mLDAC or DAC were assessed by an adaptation of the modified 2003 International Working Group Criteria as follows [32]: (1) CR as <5% BM blasts with full PB recovery (defined as neutrophil count of ≥1.0 × 10^9^/l and platelet count of ≥100 × 10^9^/l), (2) CRi and CRp as CR without full PB, (3) PR and mR as ≥50% decrease of BM blasts to 5–25% or <5% BM blasts with an Auer rod with and without full PB recovery, respectively. CRc and ORR were designated as CR + CRi + CRp, and CRc + PR + mR, respectively. In addition, relapse and disease progression were defined as the reappearance of PB or BM blasts after achieving CRc and as ≥50% increase of PB blasts and ≥25% BM blasts over baseline after treatments, respectively [13]. The duration of responses was calculated as the number of days from first achieving the corresponding response to relapse or disease progression.

Cytogenetic risk at diagnosis was categorized based on the United Kingdom Medical Research Council classification [33]. The comorbidity of patients was assessed by HCT-CI [34]. Evaluation of toxicity was performed according to the National Cancer Institute Common Toxicity Criteria for Adverse Events, Version 3.0 [35].

### Statistical analysis

The primary objective of this study was to compare the major outcomes, including response and OS rates between the mLDAC and DAC group. In addition, the incidences of clinically relevant toxicity, relapse and disease progression, and duration of responses were compared between the two groups. All time-dependent parameters were calculated from the day of the first administration of mLDAC or DAC. Continuous and categorical variables are described as medians with ranges and counts with relative frequencies, respectively. Baseline demographic and clinical characteristics of patients were compared using the independent two sample *t*-test for continuous variables and the χ^2^ or Fisher’s exact test for discrete ones. The OS rate was compared using the log-rank test, following estimates by the Kaplan-Meier method. The prognostic significance of covariates was determined using the Cox proportional hazards model for OS rate and the logistic regression model for response rates. Meanwhile, incidences of relapse and disease progression were calculated using the cumulative incidence estimates and compared using Grey’s test. The therapeutic regimen as a main interest of our study was included in all steps of model building. However, the period of administration was excluded because of strong ordinal interaction with the therapeutic regimen. Factors were considered significant if they had an associated *P* < 0.05 as determined by the likelihood ratio test, using two-tailed significance. We also validated our results by constructing a propensity-score matching cohort to alleviate any confounding effects of measured covariates between the mLDAC and the DAC groups that had an unbalanced distribution. The propensity score for each individual patient was calculated by using a logistic regression model, fitted for a therapeutic regimen by the variables which were distributed unequally or significantly affected major outcomes (age, HCT-CI, platelet count, LDH and cytogenetic risk). Subsequently, one-to-one matched groups were created by nearest-neighbor matching without replacement. Data were analyzed in July 2016 using R version 3.3.0 (R Foundation for Statistical Computing, Vienna, Austria).

## SUPPLEMENTARY MATERIALS TABLES


