# Supplementary material for: Comparison of the modified low-dose cytarabine and etoposide with decitabine therapy for elderly acute myeloid leukemia patients unfit for intensive chemotherapy
Source: Oncotarget. 2017 Dec 23;9(5):5823–33. doi: 10.18632/oncotarget.23629 (PMC5814177; doi:10.18632/oncotarget.23629)
Supplement: Supplementary file 1 [file oncotarget-09-5823-s001.pdf]

Comparison of the modified low-dose cytarabine and etoposide with decitabine therapy for elderly acute myeloid leukemia patients unfit for intensive chemotherapy

SUPPLEMENTARY MATERIALS

Supplementary Table 1: Univariate analysis of factors affecting CR, CRc, ORR, and OS rates

	CR	*P*	CRc	*P*	ORR	*P*	OS	*P*
	Rate		Rate		Rate		Rate at 1 yr	
Therapeutic regimen								
mLDAC vs. DAC	46.8 % vs. 19.0%	<0.01	50.6% vs. 23.8%	<0.01	55.8% vs. 40.5%	0.16	44.2% vs. 40.7%	0.35
Age at diagnosis								
≤70 yrs vs. >70 yrs	45.5% vs. 29.7%	0.11	49.1% vs. 34.4%	0.15	61.8% vs. 40.6%	0.04	55.5% vs. 32.5%	<0.01
ECGO PS score								
<2/≥2	30.8% vs. 40.0%	0.44	33.3% vs. 45.0%	0.31	43.6% vs. 53.8%	0.40	38.8% vs. 44.7%	0.31
HCT-CI								
<3 vs. ≥3	41.6% vs. 28.6%	0.23	46.8% vs. 31.0%	0.14	55.8% vs. 40.5%	0.16	48.6% vs. 32.8%	0.05
Disease etiology								
De novo vs MRC or secondary	38.5% vs. 30.4%	0.63	42.7% vs. 34.8%	0.65	53.1% vs. 39.1%	0.33	45.2% vs. 34.2%	0.21
WBC count								
$<10.0 \times 10^9$/l vs $\geq 10.0 \times 10^9$/l	36.5% vs. 37.5%	1.00	39.7% vs. 42.9%	0.87	52.4% vs. 48.2%	0.79	42.5% vs. 43.8%	0.28
Hemoglobin								
≤8.0 g/dl vs 8.0 g/dl	38.5% vs. 35.5%	0.87	40.4% vs 41.9%	1.00	49.1% vs. 51.6%	0.93	41.1% vs. 44.7%	0.91
Platelet count								
$<50 \times 10^9$/l vs 50×10^9/l	40.3% vs. 33.3%	0.55	45.2% vs. 36.8%	0.46	53.2% vs. 47.4%	0.65	44.8% vs. 41.2%	0.95
PB blast								
<30% vs ≥30%	37.8% vs. 36.8%	1.00	43.5% vs. 38.6%	0.72	56.5% vs. 43.9%	0.23	45.6% vs. 40.3%	0.96
BM blast								
<30% vs ≥30%	50.0% vs. 35.2%	0.44	50.0% vs. 40%	0.67	64.3% vs. 48.6%	0.41	45.9% vs. 42.5%	0.99
LDH								
≤UNL/>UNL	36.0% vs. 37.2%	1.00	40.0% vs. 41.5%	1.00	44.0% vs. 52.1%	0.62	40.0% vs. 43.8%	0.58
Cytogenetic risk								
Favorable or Intermediate vs Adverse	38.2% vs. 29.4%	0.59	43.1% vs. 29.4%	0.43	52.0% vs. 41.2%	0.58	46.4% vs. 23.5%	0.10

CR = complete remission; CRc = composite complete remission; ORR = overall response; OS = overall survival; mLDAC = modified low-dose cytarabine; DAC = decitabine; ECOG PS = the Eastern Cooperative Oncology Group performance statu; HCT-CI = the hematopoietic cell transplant co-morbidity index; MRC = myelodysplasia-related change; PB = peripheral blood; BM = bone marrow; LDH = lactate dehydrogenase; UNL = upper normal limit.

Supplementary Table 2: Outcomes of patients who received mLDAC as a salvage treatment

UPN	Admitted cycle	Group	Response	Relapse	OS	OS duration*
1	1	mLDAC	NR	NA	Dead	6.9
2	3	mLDAC	NR	NA	Dead	3.3
3	1	mLDAC	NR	NA	Dead	10.2
4	1	mLDAC	NR	NA	Dead	9.4
5	1	mLDAC	NR	NA	Dead	1.1
6	2	mLDAC	NR	NA	Dead	7.6
7	4	DAC	NR	NA	Dead	9.6
8	2	DAC	CR	Yes	Dead	7.5
9	1	DAC	NR	NA	Dead	0.9
10	1	DAC	NR	NA	Dead	9.5
11	1	DAC	mR	Yes	Dead	5.2
12	1	DAC	NR	NA	Dead	2.2
13	5	DAC	CR	No	Alive	8.4
14	1	DAC	CR	Yes	Alive	3.7
15	2	DAC	CR	No	Alive	5.2
16	4	DAC	NR	NA	Alive	3.5
17	1	DAC	CRi	No	Alive	3.3
18	1	DAC	CR	No	Alive	1.3

UPN = unique patient number; OS = overall survival; mLDAC = modified low-dose cytarabine; NR = no response; NA = not available; DAC = decitabine; CR = complete remission; PR = partial remission; CRi = incomplete complete remission
*calculated from date of mLDAC administration as a salvage treatment to any cause of death or last follow-up.

Supplementary Table 3: Outcomes of patients who received abbreviated ICTx. as a salvage treatment

UPN	Abbreviated ICTx. regimen	Group	Response	Relapse	OS	OS duration*
1	MTZ (6 mg/m^2)/ARA (30 mg) 3/14	mLDAC	NR	NA	Dead	3.1 mo
2	MTZ (6 mg/m^2)/ARA (20 mg)/VP (100 mg) 3/14/14	mLDAC	NR	NA	Dead	3.1 mo
3	MTZ (6 mg/m^2)/ARA (40 mg)/VP (100 mg) 2/5/5	mLDAC	CR	No	Dead	40.1 mo
4	IDA (12 mg/m^2)/BHAC (300 mg/m^2) 3/5	mLDAC	NR	NA	Dead	3.2 mo
5	IDA (12 mg/m^2)/BHAC (300 mg/m^2) 3/5	mLDAC	CRp	Yes	Alive	79.2 mo
6	IDA (12 mg/m^2)/BHAC (300 mg/m^2) 3/5	mLDAC	CRi	Yes	Dead	21.6 mo
7	IDA (12 mg/m^2)/BHAC (300 mg/m^2) 3/5	mLDAC	NR	NA	Dead	1.1 mo
8	CLO (40 mg/m^2)/ARA (1 g/m^2) 5/5	mLDAC	CR	Yes	Dead	8.0 mo
9	FLANG4	mLDAC	CRp	No	Dead	5.2 mo
10	MTZ (12 mg/m^2)/ARA(2 g/m^2) 3/5	mLDAC	NR	NA	Dead	2.1 mo
11	MTZ (12 mg/m^2)/ARA(2 g/m^2) 3/5	mLDAC	CR	No	Dead	12.1 mo
12	MTZ (12 mg/m^2)/ARA(2 g/m^2) 3/5	DAC	CR	No	Alive	16.9 mo
13	MTZ (12 mg/m^2)/ARA(2 g/m^2) 3/5	mLDAC	CR	No	Alive	16.9 mo

UPN = unique patient number; ICTx. = intensive chemotherapy; OS = overall survival; MTZ = mitoxantrone; ARA = cytarabine; mLDAC = modified low-dose cytarabine; CLO = clofarabine, FLANG4 = granulocyte colony-stimulating factor (300 μg for 1 day) and fludarabine (30 mg/m^2) + cytarabine (1 g/m^2) + mitoxantrone (10 mg/m^2) for 4 days; VP = etoposide; IDA= idarubicin; BHAC = N^4-behenoyl-1-β-D-arabinofuranosyl cytosine; NR = no response; NA = not available; CR = complete remission; CRp = complete remission without platelet recovery; CRi = incomplete complete remission; DAC = decitabine.
*calculated from date of abbreviated ICTx. administration as a salvage treatment to any cause of death or last follow-up.

Supplementary Table 4: Separate univariate analysis of factors affecting CR, CRc, ORR, and OS rates, respectively for the mLDAC and the DAC groups

Factors	mLDAC group	*P*	DAC group	*P*
CR rate				
Age at diagnosis				
≤70 yrs/>70 yrs	51.4% vs. 42.5%	0.44	33.3% vs. 8.3%	0.06
CRc rate				
Age at diagnosis				
≤70 yrs/>70 yrs	54.1% vs. 47.5%	0.57	38.9% vs. 12.5%	0.07
OR rate				
Age at diagnosis				
≤70 yrs/>70 yrs	58.5% vs. 52.8%	0.61	61.1% vs. 25.0%	0.02
HCT-CI				
<3 vs. ≥3	62.7% vs. 42.3%	0.09	42.3% vs. 37.5%	0.76
OS rate (at 1 yr)				
Age at diagnosis				
≤70 yrs/>70 yrs	51.4% vs. 37.5%	0.03	63.6% vs. 23.8%	0.01
ECOG performance status				
<2 vs. ≥2	48.8% vs. 38.9%	0.03	41.4% vs. 42.1%	0.32
HCT-CI				
<3 vs. ≥3	51.0% vs. 30.8%	0.07	42.9% vs. 37.5%	0.34

CR = complete remission; OR = overall response; HCT-CI = the hematopoietic cell transplant co-morbidity index; MRC = myelodysplasia-related change; OS = overall survival; ECOG = the Eastern Cooperative Oncology Group performance status.
[*]In this table, only factors with $P < 0.10$ in univariate analysis are shown.
